# Detailing Early Shoot Growth Arrest in Kro-0 x BG-5 Hybrids of *Arabidopsis thaliana*

**DOI:** 10.1093/pcp/pcad167

**Published:** 2023-12-28

**Authors:** Katelyn Sageman-Furnas, Gustavo T Duarte, Roosa A E Laitinen

**Affiliations:** Max Planck Institute of Molecular Plant Physiology, Am Mühlenberg 1, Potsdam-Golm 14476, Germany; Department of Biology, Duke University, Durham, NC 27008, USA; Max Planck Institute of Molecular Plant Physiology, Am Mühlenberg 1, Potsdam-Golm 14476, Germany; Belgian Nuclear Research Centre (SCK CEN), Unit for Biosphere Impact Studies, Boeretang 200, Mol 2400, Belgium; Max Planck Institute of Molecular Plant Physiology, Am Mühlenberg 1, Potsdam-Golm 14476, Germany; Organismal and Evolutionary Research Programme, Faculty of Biological and Environmental Sciences, Viikki Plant Science Centre, University of Helsinki, Helsinki 00014, Finland

**Keywords:** *Arabidopsis thaliana*, DYNAMIN-RELATED PROTEIN 3B, Hybrid incompatibility, Shoot growth

## Abstract

Shoot growth directly impacts plant productivity. Plants adjust their shoot growth in response to varying environments to maximize resource capture and stress resilience. While several factors controlling shoot growth are known, the complexity of the regulation and the input of the environment are not fully understood. We have investigated shoot growth repression induced by low ambient temperatures in hybrids of *Arabidopsis thaliana* Kro-0 and BG-5 accessions. To continue our previous studies, we confirmed that the Kro-0 allele of *DYNAMIN-RELATED PROTEIN 3B* causes stunted shoot growth in the BG-5 background. We also found that shoot growth repression was most pronounced near the apex at a lower temperature and that the cells in the hybrid stem failed to elongate correctly. Furthermore, we observed that shoot growth repression in hybrids depended on light availability. Global gene expression analysis indicated the involvement of hormones, especially strigolactone, associated with the dwarf phenotype. Altogether, this study enhances our knowledge on the genetic, physiological and environmental factors associated with shoot growth regulation.

## Introduction

Shoot growth plays a vital role in plant productivity and survival. It is essential for the development of reproductive organs and for transporting water and nutrients throughout the plant. Shoots are adaptive to their environment and can grow toward light, increasing photosynthesis. In agriculture, shorter stems that allow higher yield relative to total crop biomass, ensure upright growth and provide resilience to wind and rain are desired (Mathan et al. 2016). Understanding the different genetic, molecular and physiological factors controlling shoot growth offers valuable information to engineer stem growth for future environments.

Shoot growth is initiated in the shoot apical meristem and regulated by the coordinated action of hormones, mainly auxin, brassinosteroids, gibberellins and strigolactones (Rameau et al. 2015, Shi and Vernoux 2022). Shoot can be divided into internodes, which are the areas between the sideshoots. The growth of the internodes begins with cell proliferation, which is highest near the apex, and after that, cell expansion. Shoot growth regulation starts already in the meristem, and many meristem mutants are known to have reduced shoot growth (Han et al. 2020). Later on, cell wall mechanics that is regulated by organ boundary genes restricts and steers the growth of expanding cells during shoot growth (Wang et al. 2018).

Quantitative growth-related traits, including shoot growth, are genetically complex and influenced by several smaller-effect genes. While those genes with large effects have been easier to identify, we still lack information on the variety of the smaller-effect ones influencing growth-related traits. Hybrid-specific phenotypes are a powerful tool to uncover the potential role of genes involved in growth through epistasis. Interestingly, several hybrids have a temperature-dependent effect on shoot growth (Smith et al. 2011, Chae et al. 2014, Alhajturki et al. 2018, Sageman-Furnas et al. 2022). In the most common cases, dwarfism is a trade-off of the strong association of the interacting loci or alleles of the same gene in disease resistance (Chae et al. 2014).

Yet, autoactivation of disease resistance is not the only factor resulting in altered shoot growth in hybrids. It has been shown that in the case of *Arabidopsis thaliana* Sha x Lag2-2 hybrids, allelic interaction of the *OUTGROWTH ASSOCIATED KINASE* gene results in stunted growth of the stem and largely unorganized shoot branching (Smith et al. 2011, Sageman-Furnas et al. 2022). In another case, an F_1_ hybrid between two accessions, Krotzenburg-0 (Kro-0) and BG-5, shows temperature-dependent altered shoot growth compared to its parents (Alhajturki et al. 2018). When the hybrid is grown at a lower temperature (<21°C), its primary shoot is shorter and produces more lateral shoots than in the parents (Alhajturki et al. 2018). In higher temperatures, the hybrid resembles the parents. Four genes, *At2g14100* and *At3g61035* encoding for a cytochrome P450 family proteins CYP705A13 and CYP76C8P, respectively, *At2g14120* encoding for a dynamin-related protein (DRP3B) and *At3g60840* encoding a microtubule-associated protein 65-4 (MAP65-4), are known to be necessary for the phenotype (Alhajturki et al. 2018). Among them, *At2g14120* (*DRP3B*) and *At3g60840* (*MAP65-4*) from the Kro-0 parent were indicated as the most likely candidate genes for the F_1_ hybrid phenotype (Alhajturki et al. 2018). Out of these two, solely *DRP3B* has non-synonymous changes between the Kro-0 and BG-5 parents (Alhajturki et al. 2018). Here, as a next step toward understanding why the shoot growth is altered in these hybrids, we detail the genetic, molecular and cellular factors associated with the temperature-dependent shoot growth in the BG-5 x Kro-0 F_1_ hybrid.

Our analysis revealed that the reduced shoot growth in BG-5 x Kro-0 F_1_ hybrid was associated with the shorter cells and the growth arrest in the hybrids was strongest near the apex. In addition to temperature, the shoot growth arrest depended on the light conditions. Artificial shade overcame the effect of temperature on shoot growth at a lower temperature. The global gene expression analysis further supported a complex cross-talk of environmental, cellular components and hormones in controlling the altered growth. Furthermore, we confirmed that the Kro-0 allele of dynamin-related protein 3B (DRP3B) causes reduced stem growth when transferred to the BG-5 background. This study reports novel information on the different factors influencing plant shoot growth.

## Results

### The short stem of the hybrids is due to failure of stem elongation

**Fig. 1:**
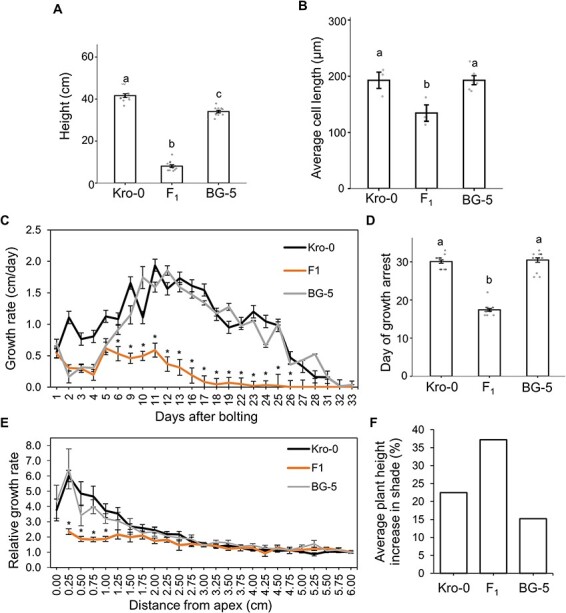
Characterization of the stem growth in the Kro-0 x BG-5 hybrid phenotype. (A) Analysis of the stem growth in the hybrids and the parent stems when grown at 17°C in mature plants after the start of senescence. Bars represent standard error (SE) of  *n*> 13 for each genotype, and different letters represent significant differences according to one-way ANOVA (*P* < 0.05). (B) Cell length analysis of mature stems. Bars represent SE of 10 measured cells for *n* > 3 for each, and different letters represent significant differences according to one-way ANOVA (*P* < 0.05). (C) Analysis of the stem growth rate in hybrids and parents grown at 17°C, beginning from bolting. Stars represent statistical significance of the F_1_ hybrid and the parents, one-way ANOVA, *n* = 16, each genotype. (D) Average day of growth arrest, counted from the bolting of the stem. Letters represent statistical significance, one-way ANOVA, *n* = 16, each genotype (E). Dot experiment representing daily 0.25-cm increments after the formation of the first silique, and stars represent significance in growth rate between the hybrid and the parents with a one-way ANOVA, *n* > 13 plants each genotype. (F) Shade experiment. Increase in height when grown under shade (R:FR ratio = 0.7) and normal (R:FR = 5–7) conditions at 17°C. Kro-0 parents had average heights of 50.98 ± 4.14 and 41.63 ± 3.03, BG-5 parents had 40.46 ± 3.06 and 35.13 ± 5.97 and the F_1_ had 11.09 ± 2.49 and 8.08 ± 2.27 under shade and normal conditions. *n* > 12 plants each genotype and condition.

The F_1_ hybrid between Kro-0 (Krotzenburg, Germany) and BG-5 (Seattle, USA) parents has altered shoot architecture with strongly reduced main shoot growth at lower temperatures <21°C (Alhajturki et al. 2018). The largest effect on stem growth was observed in the first and second internodes of the stem (Alhajturki et al. 2018, **Fig. 1A**). Cryo-scanning electron microscopy revealed that the epidermal cell lengths in the hybrids’ first and second internodes are reduced compared to parents (**Fig. 1B**). To test if the shoots grew more slowly or had early meristem arrest, we monitored stem height in hybrids and parents daily for 33 d at 17°C and 23°C starting from the bolting of the stem (**Fig. 1C**). The early growth rate of the hybrid stem was comparable to that of the BG-5 parent up to the fifth day. However, the hybrid shoot growth arrested on average 18 d after germination, while both parents continued to grow until approximately 33 d after germination (**Fig. 1C, D**). This early cessation of growth is likely not due to an earlier developmental transition because the flowering time in the hybrid at 17°C was similar to the Kro-0 parent (**Supplementary Table S1**). To identify which part of the stem was responsible for such a differential elongation, we dotted the stems in hybrid and parental plants in 0.25-cm increments after the first silique was formed and quantified the daily stem growth from the apex. While both parents grew rapidly near the apex, and the growth only ceased further away from the apex, the hybrid’s stem growth rate was relatively flat (**Fig. 1E**). This indicates that the reduced shoot growth in hybrids is due to the lack of growth signals from the meristem. Since meristem-defective mutants such as *wuschel* and *clavata* have altered meristem size (Kitagawa and Jackson 2019), we measured the meristem size of the parents and hybrids at 17°C. The meristems in the hybrid appeared normal (**Supplementary Fig. S1**), and no implications for meristem defects were observed.

### Red:far-red ratio affects the hybrid phenotype

Light availability is the main factor known to regulate shoot architecture and plant height, and the ratio between the red (R) and far-red (FR) affects shoot height (Carriedo et al. 2016). A higher amount of FR light is known to promote stem growth similar to shade conditions (Carriedo et al. 2016). We hypothesized that if the slow growth reflects a higher stem tolerance to FR of the hybrid compared to parents under cooler conditions, an increased amount of FR light would resume the normal stem growth in the hybrids. To test this, we compared the stem growth of parents and hybrids under normal (R:FR = 5–7) and shaded conditions (R:FR = 0.7) at 17°C. Under shaded conditions, the hybrid showed stronger response to the light conditions than the parents (**Fig. 1F**). Kro-0 and BG-5 increased their height approximately 22% and 15%, respectively, while the F_1_ hybrid height increased nearly 40% (**Fig. 1F**), showing that light and particularly the ratio of R:FR light influence the shoot growth phenotype in the hybrid.

### Dynamin-related protein 3B causes reduced stem height in the BG-5 background

**Fig. 2:**
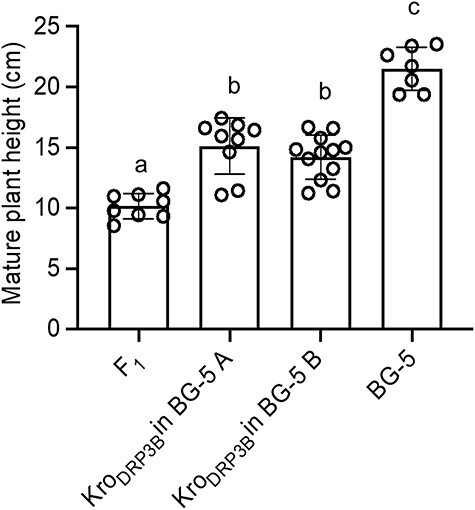
DRP3B causes reduced stem height in the BG-5 background. Kro-0 DRP3B causes reduced stem growth when introduced to BG-5. The final heights of transgenic lines containing the Kro-0 allele of *DR3PB* were analyzed growing them at 17°C. *N* > 9 T2 plants confirmed using PCR for the presence of transgene for each transformed line. Bars represent SE.

From the four genes necessary for the altered stem growth in F_1_ hybrid, *DRP3B* was found to be the most likely causal candidate for the F_1_ phenotype (Alhajturki et al. 2018). To investigate whether the Kro-0 allele of *DRP3B* is sufficient to induce the phenotype in the BG-5 background, we synthesized the Kro-0 allele of the *DRP3B* gene and transferred it to the BG-5 parent. BG-5 plants transformed with *DRP3B*_Kro-0_ had significantly reduced shoot growth at 17°C compared to the BG-5 wild type (**Fig. 2**). To confirm that the phenotype is due to epistatic interaction requiring both the Kro-0 allele of *DR3PB* and the BG-5 allele in chromosome 2, we tested whether the single mutant of DRP3B in the Col-0 background showed repressed shoot growth. The shoot growth of the single mutant in the Col-0 background did not differ from the wild type (**Supplementary Fig. S2**). This confirmed that the Kro-0 allele of *DRP3B* causes reduced shoot height in combination with the BG-5 genetic background in the F_1_ hybrids.

### Gene expression associated with reduced stem growth

**Fig. 3:**
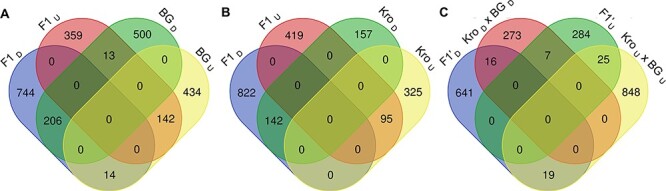
Global expression analysis of temperature-responsive genes among BG-5, F_1_ hybrid and Kro-0. (A) Venn diagram representing unique and shared up (U)- and down (D)-regulated genes between the F_1_ (**Supplementary Table S2**) and BG-5 (**Supplementary Table S3**). (B) Venn diagram representing unique and shared up- and downregulated genes between the F_1_ (**Supplementary Table S2**) and Kro-0 (**Supplementary Table S4**). (C) Venn diagram representing unique and shared up- and downregulated genes between the F_1_-specific temperature-responsive genes and genes that are inversely regulated in the hybrid in comparison to either BG-5 and Kro-0 and the temperature-responsive genes differentially regulated between BG-5 and Kro-0.

Next, we aimed to understand which regulatory pathways are associated with reduced shoot growth in the hybrids at lower temperatures. We performed a global gene expression analysis comparing a pooled sample of mature first and second internodes of the parents and the hybrid stems grown at 17°C and 23°C. A total of 1,478, 1,309 and 719 differentially expressed genes (DEGs) were identified in the F_1_, BG-5 and Kro-0 parents in response to temperature, respectively (**Supplementary Tables S2–S4**), considering log_2_ fold change (FC) ≥ |1.0| and false discovery rate (FDR) < 0.05 as threshold. Of these, 99 genes were commonly regulated among the three genotypes (**Supplementary Fig. S3, Supplementary Table S5**). Overall, the F_1_ shared a higher number of DEGs with the BG-5 parent (21.4% and 27.6% of F1’s down- and upregulated DEGs, respectively; **Fig. 3A**) than with Kro-0 (14.7% and 18.5% for the down- and upregulated DEGs, respectively; **Fig. 3B**). Because the hybrid only shows the phenotype when grown at 17°C and resembles the parents when grown at 23°C, we first filtered those temperature-responsive DEGs that were specifically differentially expressed in the hybrid at 17°C in comparison to 23°C or that showed an opposite expression pattern between the hybrid and the parents. Next, considering that the parents were phenotypically similar when grown at 17°C, we removed DEGs commonly identified in the hybrid and either of the parents between 17°C and 23°C from the hybrid-specific DEGs (**Fig. 3C**). This resulted in 925 hybrid-specific temperature-responsive genes, of which 284 are upregulated and 641 are downregulated (**Fig. 3C**; **Supplementary Table S6**).

Gene ontology (GO) analysis of the upregulated DEGs revealed an enrichment in processes related to hormone response such as salicylic acid, abscisic acid (ABA), ethylene, RNA biogenesis regulation and cell differentiation/organism development (**Supplementary Fig. S4** and **Supplementary Table S7**). Among the molecular functions, the control of transcription (binding activity and transcription factor activity) and transmembrane transport were enriched. No cellular components were enriched among the upregulated DEGs. The downregulated genes included catabolic and cell wall–related processes (**Supplementary Fig. S5** and **Supplementary Table S8**). In addition, our analysis showed a clear enrichment for photosynthesis- and light-related processes and included chloroplast elements among the most enriched cellular components (**Supplementary Fig. S5B** and **Supplementary Table S8**). Among molecular functions, oxidoreductase activity was enriched. Interestingly, morphogenesis of a branching structure was among the hybrid-specific downregulated terms. In this group, four genes (*AT4G32810, AT1G03055, AT2G44990* and *AT2G26170*) are involved in carotene metabolism and strigolactone biosynthesis.

### Co-expression analysis of the direct and indirect roles of DRP3B

We showed that *DRP3B* was responsible at least for the reduced stem length in the hybrid (**Fig. 2**), but it was not among the differentially regulated genes in any of the comparisons (**Supplementary Tables S2–4**). We reasoned that since the different expression of *DRP3B* was not linked with the F_1_ hybrid phenotype, the phenotype might be the outcome of the downstream effects of *DRP3B*. To investigate this possibility, we performed a co-expression analysis for the *DRP3B* to evaluate if the co-expressed genes showed hybrid-specific temperature response in our RNA-seq analysis (**Supplementary Table S9**). However, none of the co-expressed genes showed such a pattern.

**Fig. 4:**
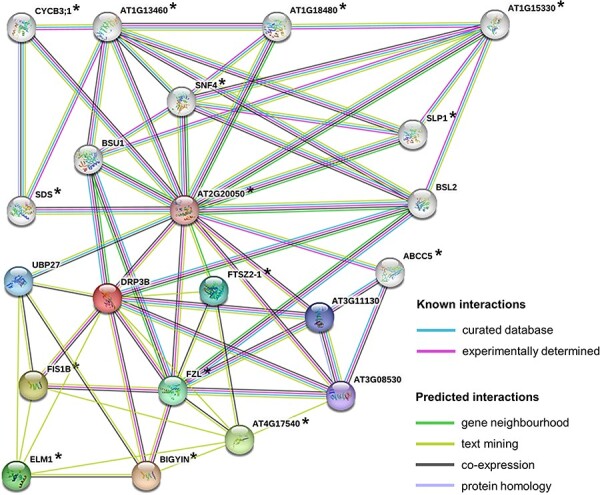
DRP3B protein co-expression network. The figure represents proteins known and predicted to interact with DRP3B according to the STRING database (Szklarczyk et al. 2019) (https://string-db.org/). The proteins marked with an asterisk in their gene co-expression network elements were differentially regulated in the hybrid in temperature-specific manner (**Supplementary Tables S10–S12**). The gene co-expression network of each of DRP3B’s interacting proteins (**Supplementary Table S10)** was retrieved from ATTED-II database (https://atted.jp/).

Next, considering that a non-synonymous change differentiates Kro-0 and BG-5 alleles of *DRP3B*, we hypothesized that the altered hybrid phenotype caused by the *DRP3B* allele inherited from Kro-0 could influence protein interaction and result in altered interactions at the protein level. Therefore, we identified 20 proteins that are known to interact with DRP3B, and we analyzed the co-expression network for each of them. This resulted in a panel of candidate genes indirectly contributing to the observed phenotype. Interestingly, from the 20 proteins that directly or indirectly interact with DRP3B (**Fig. 4**), 15 were co-expressed with several genes that were specifically up- or downregulated in the F_1_ hybrid in a temperature-dependent manner (**Supplementary Table S10**). For at least six of them, the co-regulated network at least partly explained the GO enrichment observed in the F_1_, such as control of transcription, response to ABA and transmembrane transport activity among the upregulated genes (**Supplementary Table S11; Supplementary Fig. S6**), and photosynthesis- and light-related elements among the downregulated genes (**Supplementary Table S12; Supplementary Fig. S7**). Indeed, among the genes that were part of those uniquely expressed in the F_1_ hybrid, there was an interesting overlap with the co-expression network of *AT4G17540, FTSZ2-1, FZL* and *SLP1* that points to chloroplast-related responses (**Supplementary Table S10**). Furthermore, *ABCC5* and *SLP1* were co-expressed with two central strigolactone biosynthesis pathway elements, *MAX1* (*CYP711A1*) and *D27*, respectively, which are known to reduce shoot growth and lead to a bushy phenotype in the case of disruption (Bennett et al. 2016) In the hybrid, these strigolactone-related genes were downregulated (**Supplementary Tables S6 and S10**). In addition, *ABCC5* is also co-expressed with a brassinosteroid gene that has a dwarf mutant phenotype (*STE1*). These results further support the existing evidence of the underlying role of hormone interaction in the altered shoot growth in hybrids.

## Discussion

We have detailed, using genetic, molecular and cellular approaches, temperature-dependent shoot growth arrest in the F_1_ hybrid between Kro-0 and BG-5 accession of *A. thaliana*. We showed, that in addition to temperature, the hybrid phenotype depends on light availability. Hybrids increased their height more than the parents under increased FR light conditions, even at lower temperatures (**Fig. 1**). We further demonstrated that the reduced shoot growth in the F_1_ hybrid in comparison to its parents was due to arrest of shoot growth resulting in shorter epidermal cells. We observed that the temperature-repressed growth of the hybrid stem was the strongest near the apex. Although this is indicative of meristem arrest, the lack of differences in meristem size (**Supplementary Fig. S1**) made us dismiss the possible role of meristem defect in causing the repressed shoot growth.

To get insights into the processes underlying the repressed shoot growth in hybrids, a global gene expression analysis comparing the hybrid and parent stems grown at different temperatures was performed. Interestingly, genes involved in carotene metabolism and strigolactone biosynthesis were among the hybrid-specific downregulated genes, including *MAX1* (*CYP711A1; AT2G26170*), *MAX3* (*ATCCD7; AT2G44990*), *MAX4* (*ATCCD8; AT4G32810*) and *D27* (*AT1G03055*) (**Supplementary Tables S6 and S10**). Furthermore, the strigolactone negative regulator SMXL6 (Soundappan et al. 2015) was among the hybrid-specific upregulated genes (**Supplementary Table S6**). Similar to our F_1_ hybrid (**Fig. 1**), many of the strigolactone mutants exhibit reduced height (Brewer et al. 2013). In addition, strigolactone is known to affect auxin transport via auxin export protein PIN1 (Shinohara et al. 2013). In line with this, we found that several auxin-responsive genes (e.g. *IAA1/AXR5, IAA18* and *IAA20*) and the auxin efflux carrier *PILS5* specifically downregulated in the hybrid at 17°C (**Supplementary Table S6**). Taken together, these results suggest that the repressed shoot growth in the F_1_ hybrids involves a complex cross-talk of plant hormones, especially auxin and strigolactones.

We have previously shown that shoot growth arrest in the F_1_ hybrid between Kro-0 and BG-5 is due to interaction between a chromosome 2 locus from Kro-0 and chromosome 3 locus from BG-5 (Alhajturki et al. 2018). Furthermore, four genes were found to be necessary for the F_1_ phenotype, namely, *At2g14120* (*DRP3B*), *At2g14100* (*CYP705A13*), *At3g61035* and *At3g60840* (*MAP65-4*) (Alhajturki et al. 2018). We have also shown that expression of *DRP3B* using an artificial microRNA strategy in the hybrid rescues the shoot growth phenotype (Alhajturki et al. 2018). In this study, by synthesizing the Kro-0 allele of *DRP3B*, we proved that Kro-0 like *DRP3B* causes reduced stem growth when transferred to the BG-5 background. However, the single mutant of *DRP3B* in the Col-0 background did not show repressed shoot growth (**Supplementary Fig. S2**). This demonstrates that the repressed shoot growth is due to epistatic interaction with the Kro-0 allele of *DRP3B* with the either of the possible partners on chromosome 3, *MAP65-4* or *At3g61035* genes, from BG-5.

In *A. thaliana*, the two redundantly acting copies of DRP3, DRP3A and DRP3B mediate the mitochondria and peroxisome fission, resulting in reduced growth (Fujimoto et al. 2009, Zhang and Hu 2009, Aung and Hu 2012, Nagaoka et al. 2017). In addition, the double mutants of *DRP3A* and *DRP3B* in the Col-0 background have slower growth than the single mutants and have a reduced number of mitochondria and peroxisomes (Zhang and Hu 2009). Another member of the dynamin-related proteins, DRP1, was found to be necessary for the correct localization of PIN1 during cytokinesis (Mravec et al. 2011). This together with our global gene expression data suggests that the interplay of DRP3B and MAP65-4 /and/or At3g61035 could influence the distribution of PIN-FORMED (PIN) auxin efflux carriers via microtubules, thereby regulating the shoot growth in the hybrids. This possibility is supported by our earlier observation of the lower abundance of indole acetic acid (IAA), an active form of auxin, in the F_1_ hybrid at lower temperatures (Alhajturki et al. 2018).

To conclude, this study suggests that the mechanism underlying growth arrest in the BG-5 x Kro-0 hybrids is a result of the interplay of altered hormone signaling in the shoot and environmental signals, including light and temperature.

## Materials and Methods

### Plant lines and growth conditions

Seeds for the *A. thaliana* accessions Kro-0 (CS1301; Krotzenburg, Germany), BG-5 (CS22345, Seattle, USA), Col-0 (CS22681, Columbia, USA) and a T-DNA insertion line targeting DRP3B (SALK_017492C) were obtained from the Nottingham Arabidopsis Stock Centre (Loughborough, United Kingdom). Seeds were stratified in 0.1% (w/v) agarose in water at 4°C in the dark and then sown onto soil. Plants were grown either under long-day (LD) conditions (16 h/8 h; 23°C/ 17°C) or short-day conditions (8 h/16 h; 23°C/17°C). For phenotyping, plants were grown at constant temperatures of 17°C or 23°C under LD conditions. To measure the daily growth of the stem from the apex, hybrid and the parents were grown with 16 replicates at 17°C. The daily growth was measured from the dots marked to the stem each day (Bencivenga et al. 2016). Under all conditions, the light intensity was 150 µE m^−2^ s^−1^ and the humidity was 60–70%. To generate F_1_ hybrids, BG-5 was used as the pollen donor. For shade experiments, Kro-0 and BG-5 parents and F_1_ plants were grown at 17°C for 5 weeks in a Percival growth chamber under LD conditions with at least 12 replicates. This experiment was repeated twice with similar results. Seedlings in individual pots were then placed in another Percival with a R to FR light ratio of 0.7 and a light intensity of 96 µmol m^−2^ s^−1^ resembling shade. Pots were randomized and watered only with exterior lights off to protect shade conditions. Under all conditions, the stem length was scored after siliques began to form.

### Microscopic analysis of the cell size

To quantify the cell length in the stems, the first and second internodes of Kro-0, F_1_ and BG-5 plants were collected into liquid nitrogen. Sections of internodes were imaged using scanning electron cryomicroscopy. Epidermal cell lengths were measured using ImageJ (**Fig. 1B**) and averaged across the parent and hybrid.

### Generation of transgenic plants

For the genomic construct of *DRP3B*, a genomic region of 7,436 bp extracted from the 1001 genome project from the Kro-0 parent was synthesized via Genewiz (Burlington, MA 01803, USA). The resulting fragment was subcloned into pCambia 1302 and transformed into BG-5 via *Agrobacterium tumefaciens* (GV3101) using the floral dip method (Clough and Bent 1998). T_0_ seeds were selected on 50 µg ml^−1^ hygromycin. Two resistant T_1_ lines were further confirmed for homozygosity, and at least 10 plants of both lines in the T_2_ generation were grown at 17°C. Stem length was measured after siliques were formed.

### Transcriptome sequencing and analysis

First and second internodes of four individuals of Kro-0, the F_1_ hybrid and BG-5 were harvested separately at 17°C and 23°C from four 5-week-old plants. RNA from each of the four replicates was isolated with the QIAGEN RNeasy Plant Mini Kit with an on-column DNase treatment before sequencing. BGI Genomics (www.bgi.com) carried out the library preparation and the sequencing.

Quality processing of RNA-seq reads was performed as previously reported (Duarte et al. 2021), using the Phred score of 30 as a cutoff. The processed files were deposited into the Sequence Read Archive (accession PRJNA915341). The transcriptome assembly and differential expression analysis were conducted on the Galaxy Europe platform (https://usegalaxy.eu/; Afgan et al. 2018). Trimmed reads of each sample were mapped to the *A. thaliana* TAIR10 reference genome (https://www.arabidopsis.org/; release 2022-08-04) and the reference GTF annotation obtained from Ensembl Plants (http://plants.ensembl.org/; release 49) using HISAT2 v.2.1.0 (Kim et al. 2015). The following parameters were set: unstranded, mate orientation (–fr), alignments were reported tailored for StringTie (–dta), and remaining parameters as default. For each HISAT2 output, transcript expression analysis was performed with StringTie v.2.1.1 (Kovaka et al. 2019) using default values but providing the reference GTF annotation (–G option) and reporting only known transcripts (–e option) for DESeq2 analysis (Love et al. 2014). Genes showing *P*-value < 0.05 after Benjamini and Hochberg correction and log_2_FC > |1| were retained as differentially expressed.

### Data analysis

DEGs overlap analysis was performed using Venn diagrams (https://bioinformatics.psb.ugent.be/webtools/Venn/) and UpSet plots, which was run in RStudio 2021.09.0 + 351 using the ‘UpSetR’ package v1.4.0. The heatmaps were plotted using the ‘ComplexHeatmap’ package v. 2.10.0 (Gu et al. 2016). GO enrichment analysis was performed using agriGO v2.0 [http://systemsbiology.cau.edu.cn/agriGOv2/ (Tian et al. 2017)] plant Singular Enrichment Analysis program with the built-in TAIR10_2017 reference as the background. DEGs that were up- or downregulated were treated separately for each specified comparison. Each full list containing all GO terms (**Supplementary Tables S7, S8, S11, and S12**) was summarized using revise +visualize gene ontology (REVIGO) v1.8.1 [http://revigo.irb.hr (Supek et al. 2011)] with similarity set to 0.7 and associated with their respective FDR values, using as reference the *A. thaliana* GO database. For the data presented in **Supplementary Tables S7 and S8**, which represent the unique hybrid-specific temperature-responsive genes, the REVIGO tree map was converted into a circular plot using CirGO (Kuznetsova et al. 2019). All graphs were further edited using Microsoft Office. Gene co-expression networks were retrieved from ATTED-II v11.1 [https://atted.jp/; (Obayashi et al. 2022)], setting the supportability to E-01 for up to 1000 co-expressed elements. Protein co-expression networks were obtained from search tool for the retrieval of interacting genes/proteins (STRING) v11.5 database [https://string-db.org/; (Szklarczyk et al. 2019)] using default parameters.

## Supplementary Material

pcad167_Supp

## Data Availability

The data generated or analyzed during the current study are available from the corresponding author on reasonable request. The RNA-seq data have been submitted to national center for biotechnology information (NCBI) sequence read archive (SRA) database with reference ID PRJNA915341 (link https://www.ncbi.nlm.nih.gov/bioproject/771496).
