# Supplementary material for: Detailing Early Shoot Growth Arrest in Kro-0 x BG-5 Hybrids of Arabidopsis thaliana
Source: Plant Cell Physiol. 2023 Dec 28;65(3):420–7. doi: 10.1093/pcp/pcad167 (PMC11020215; doi:10.1093/pcp/pcad167)
Supplement: pcad167_Supp [file pcad167_supp.zip › suppl_data/pcp-2023-e-00242-File006.pdf]

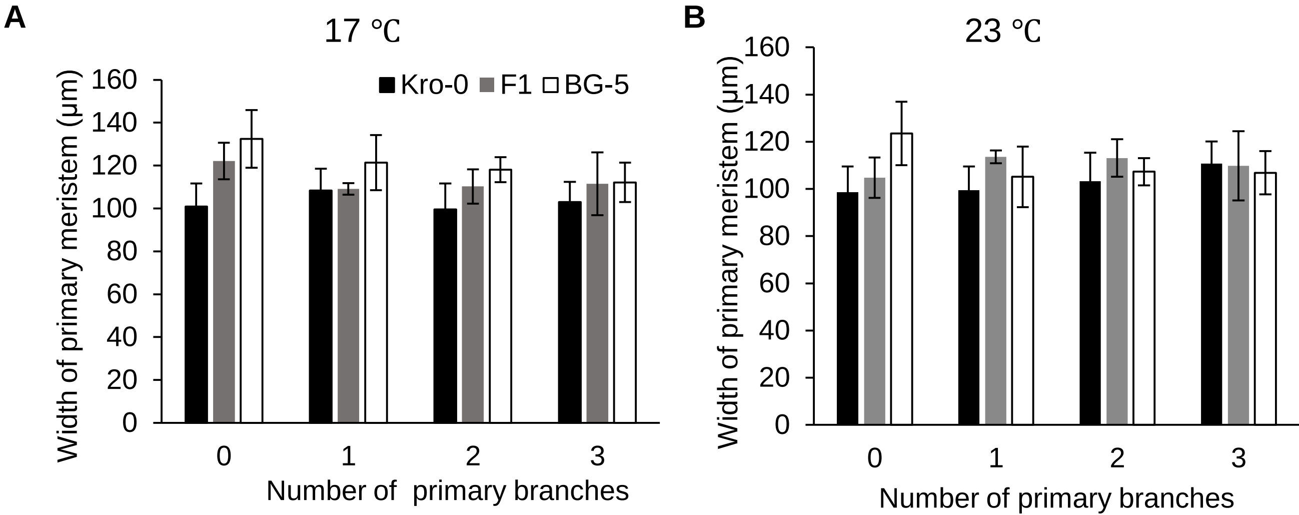


**Figure S1. Meristem size of Kro-0, the $F_1$ hybrid and BG-5. A.** Size at17°C. **B.** Size at 23°C. Meristem size was measured with ImageJ at different stages of development defined as number of branches at both 17°C and 23°C. Bars represent SD, N = 3 or 4 each genotype/treatment. No statistical differences were found.
