# Supplementary material for: Detailing Early Shoot Growth Arrest in Kro-0 x BG-5 Hybrids of Arabidopsis thaliana
Source: Plant Cell Physiol. 2023 Dec 28;65(3):420–7. doi: 10.1093/pcp/pcad167 (PMC11020215; doi:10.1093/pcp/pcad167)
Supplement: pcad167_Supp [file pcad167_supp.zip › suppl_data/pcp-2023-e-00242-File007.pdf]

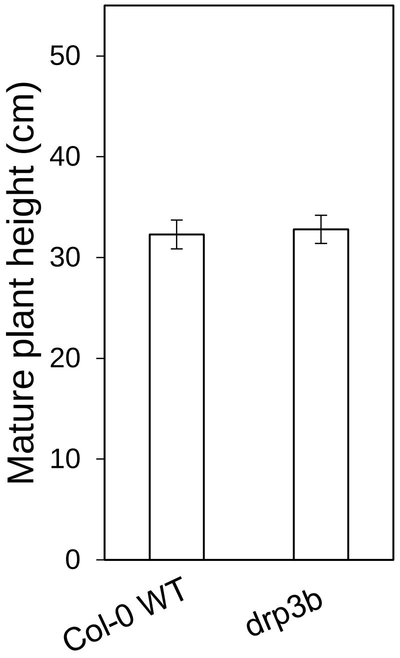

Figure S2. Loss of DRP3B in Col-0 does not affect plant height.

Final heights of Col-0 WT and a salk line targeting DRP3B (*drp3b*) grown at 17 °C were measured after siliques were formed. Bars represent SE, N > 25 each line. Bars represent SE.
