# Supplementary material for: Detailing Early Shoot Growth Arrest in Kro-0 x BG-5 Hybrids of Arabidopsis thaliana
Source: Plant Cell Physiol. 2023 Dec 28;65(3):420–7. doi: 10.1093/pcp/pcad167 (PMC11020215; doi:10.1093/pcp/pcad167)
Supplement: pcad167_Supp [file pcad167_supp.zip › suppl_data/pcp-2023-e-00242-File008.pdf]

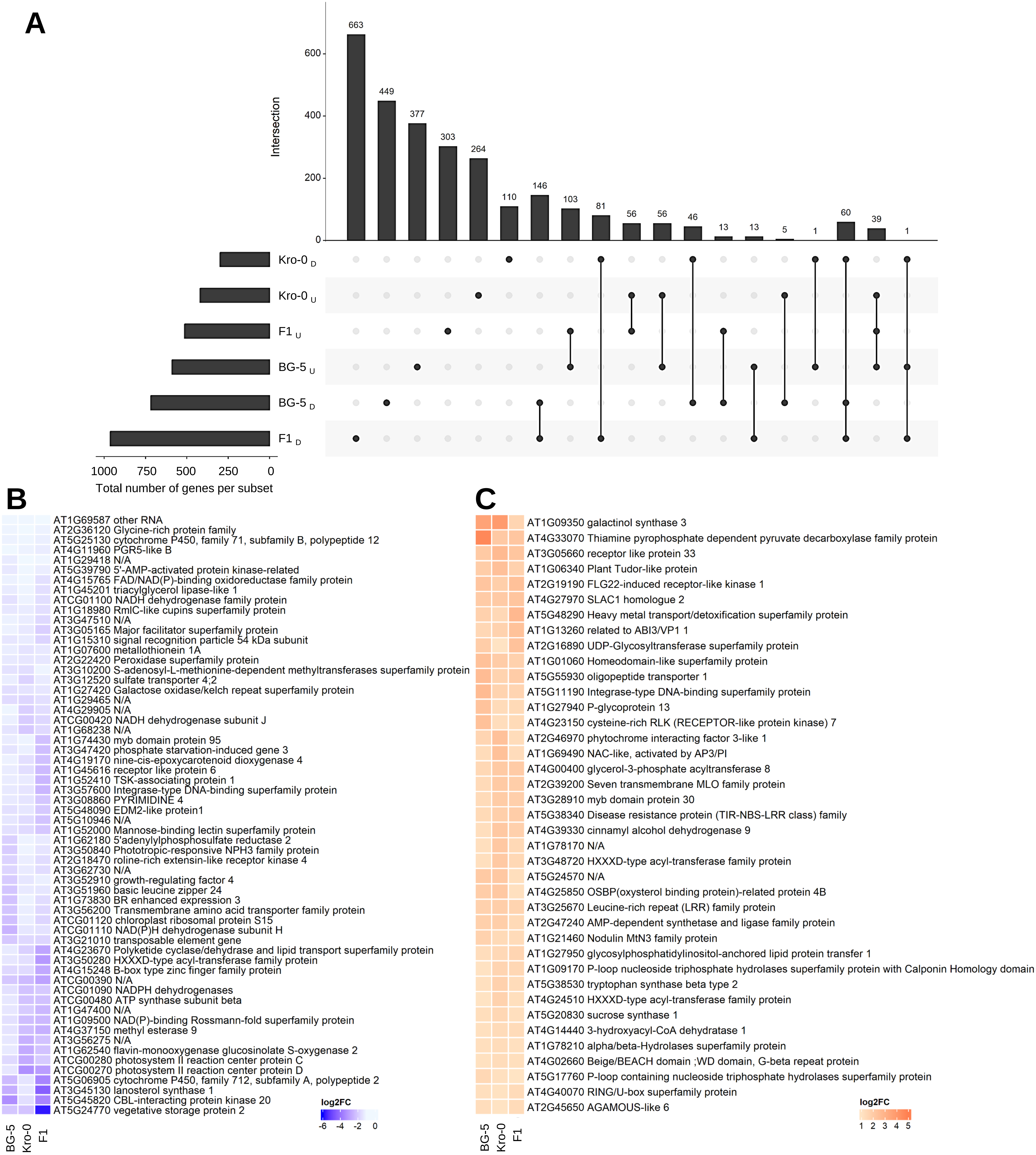

Figure S3. Comparison of the global expression profiles of BG-5, F_1 hybrid, and Kro-0 in response to temperature. A. UpSet plot representing the number of up-regulated (U) and down-regulated (D) DEGs among the three genotypes. Each column represents the set denoted by the filled-in cell underneath it. Intersections among the up- and/or down-regulated DEGs of the three genotypes are denoted by connected filled-in cells. **B.** Heatmap representing the expression pattern of the 60 genes commonly down-regulated among the three genotypes. **C.** Heatmap representing the expression pattern of the 39 genes commonly up-regulated among the three genotypes. The values represent the log_2FC of plants grown at 17°C compared to 23°C respective to each genotype.
