# Supplementary material for: Detailing Early Shoot Growth Arrest in Kro-0 x BG-5 Hybrids of Arabidopsis thaliana
Source: Plant Cell Physiol. 2023 Dec 28;65(3):420–7. doi: 10.1093/pcp/pcad167 (PMC11020215; doi:10.1093/pcp/pcad167)
Supplement: pcad167_Supp [file pcad167_supp.zip › suppl_data/pcp-2023-e-00242-File009.pdf]

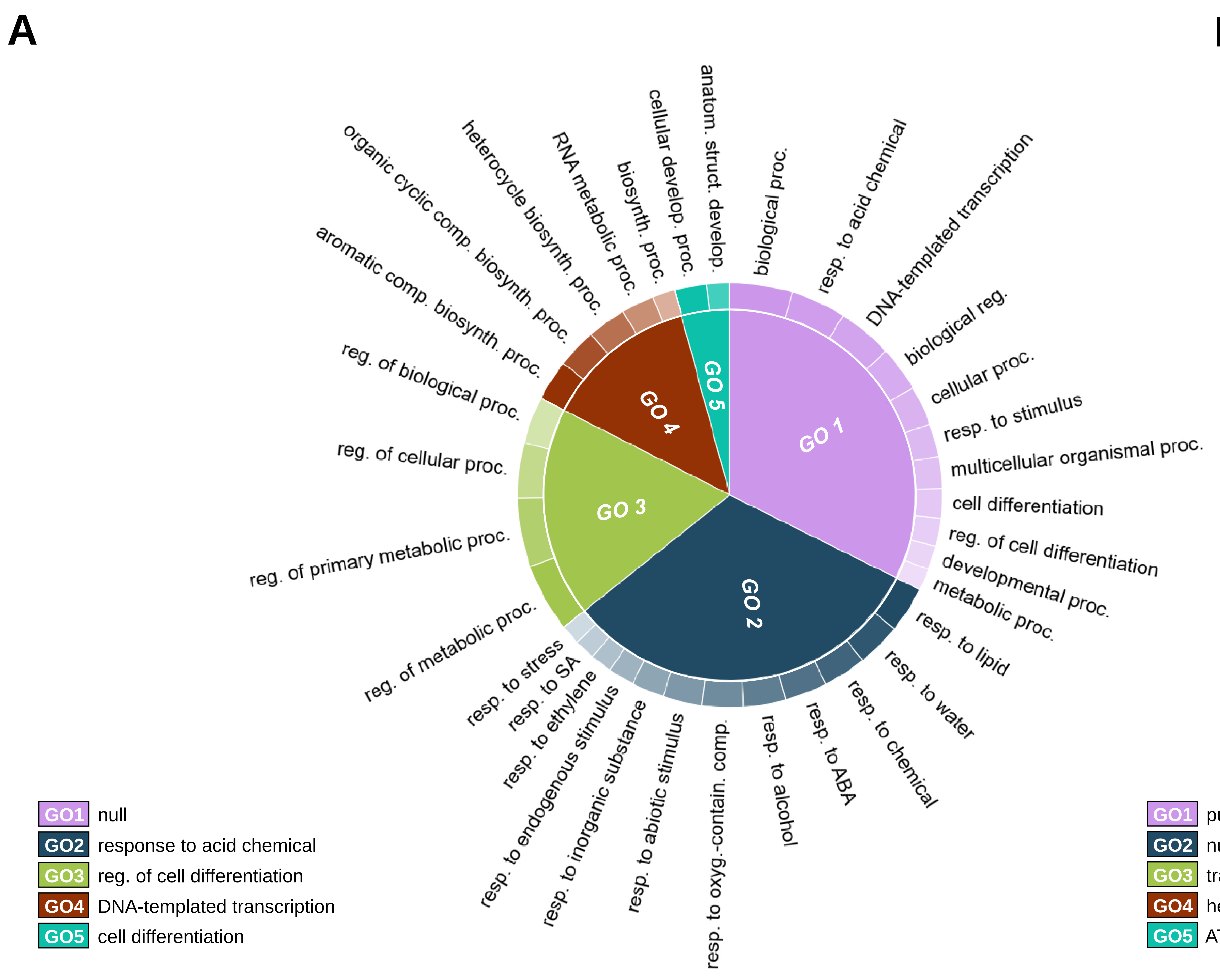

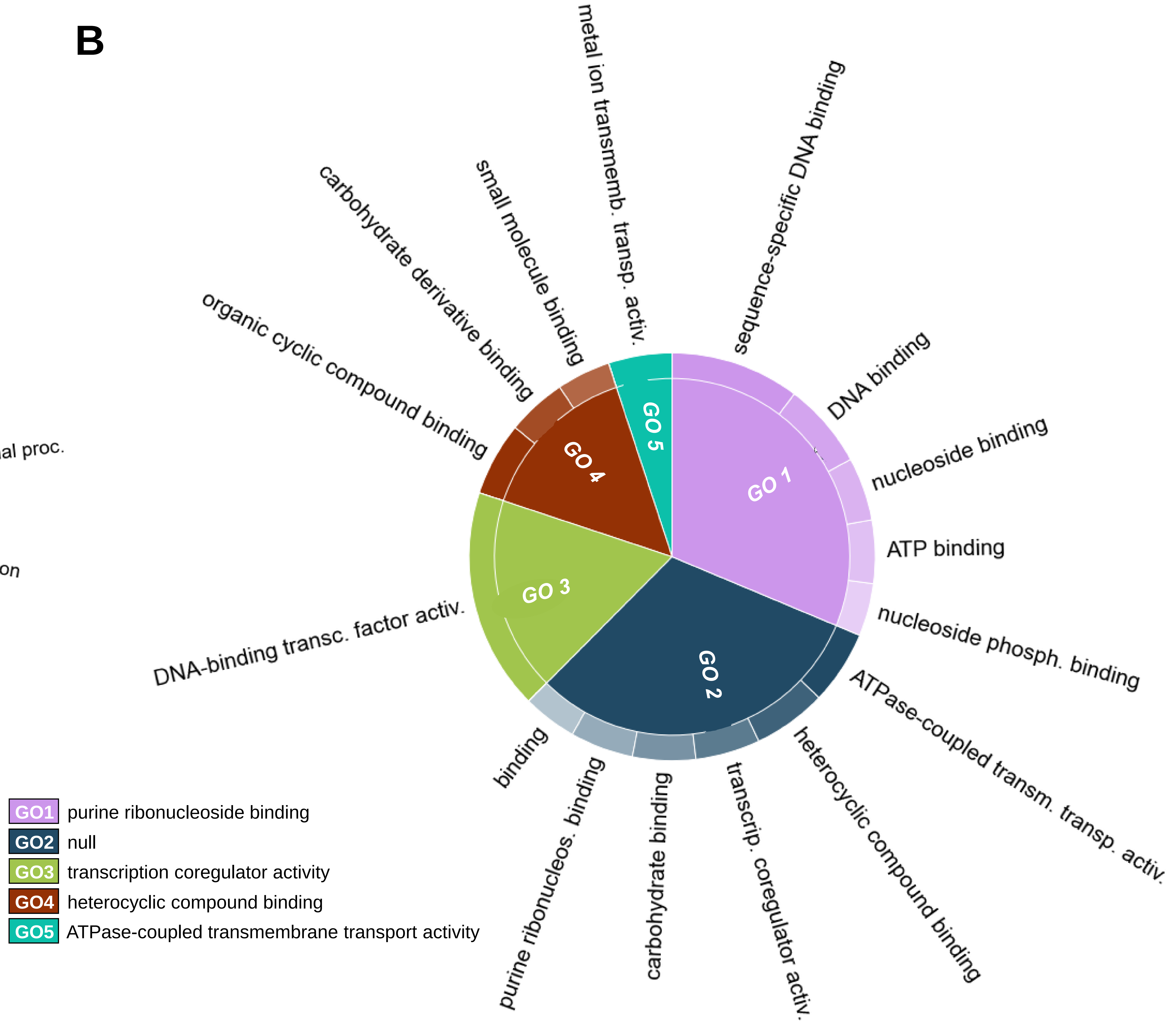

Figure S4. Enriched GO terms among the unique F_1-specific temperature-responsive up-regulated genes. A. Biological processes. **B.** Molecular functions. The inner circle represents parent GO categories, which are denoted in the legend respective to each plot. “Null” contains parent terms that represent themselves. The GO plots were created with CirGO (Kuznetsova et al. 2019), using enrichment data obtained with agriGO (Tian et al. 2017) and summarized with Revigo (Supek et al. 2011).
