# Supplementary material for: Detailing Early Shoot Growth Arrest in Kro-0 x BG-5 Hybrids of Arabidopsis thaliana
Source: Plant Cell Physiol. 2023 Dec 28;65(3):420–7. doi: 10.1093/pcp/pcad167 (PMC11020215; doi:10.1093/pcp/pcad167)
Supplement: pcad167_Supp [file pcad167_supp.zip › suppl_data/pcp-2023-e-00242-File011.pdf]

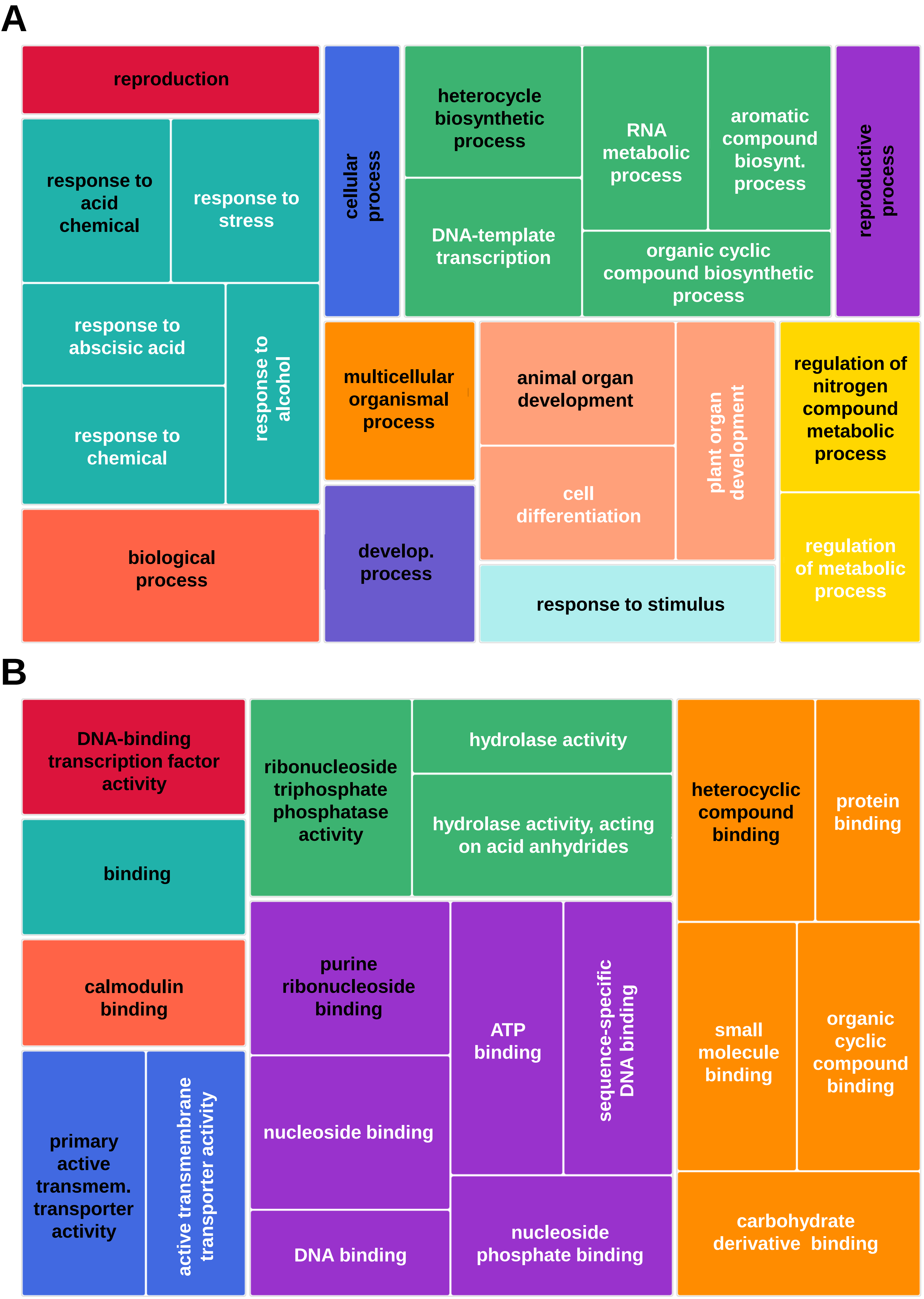

Figure S6. Summarized gene ontology enrichment plots for hybrid-specific up-regulated temperature-responsive genes within the co-expression network of DRP3B's interacting partners. A. Biological process terms. **B.** Molecular function terms. The plots were generated with Revigo using the enrichment data obtained with agriGO (Table S10). Superclusters are represented by different colors. Black labels denote the representative terms, which group those terms with similar classification. The size of the rectangles reflects the p-value of the respective enriched term among all enriched ones.
