# Supplementary material for: Detailing Early Shoot Growth Arrest in Kro-0 x BG-5 Hybrids of Arabidopsis thaliana
Source: Plant Cell Physiol. 2023 Dec 28;65(3):420–7. doi: 10.1093/pcp/pcad167 (PMC11020215; doi:10.1093/pcp/pcad167)
Supplement: pcad167_Supp [file pcad167_supp.zip › suppl_data/pcp-2023-e-00242-File012.pdf]

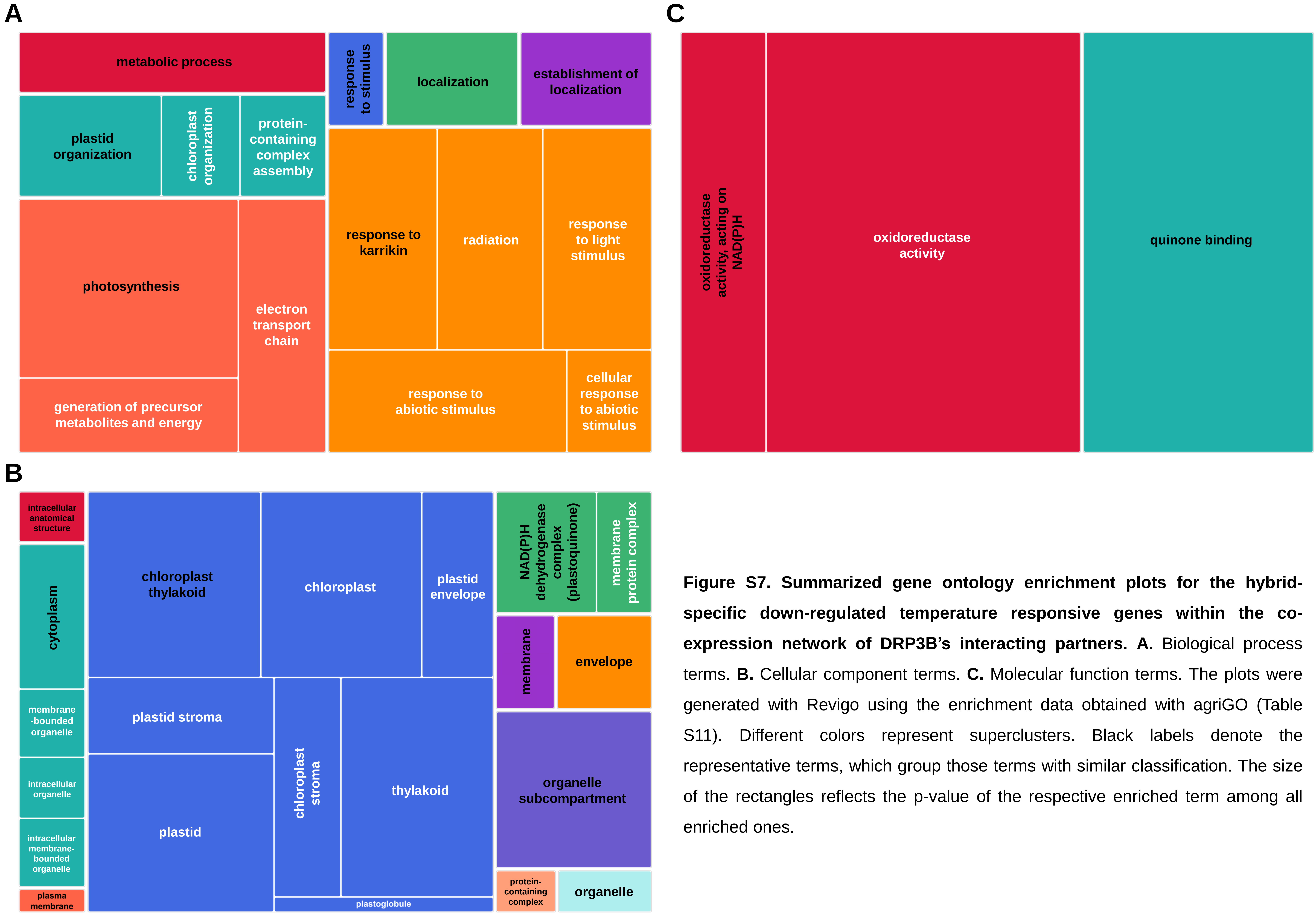

Figure S7. Summarized gene ontology enrichment plots for the hybrid-specific down-regulated temperature responsive genes within the co-expression network of DRP3B's interacting partners. A. Biological process terms. **B.** Cellular component terms. **C.** Molecular function terms. The plots were generated with Revigo using the enrichment data obtained with agriGO (Table S11). Different colors represent superclusters. Black labels denote the representative terms, which group those terms with similar classification. The size of the rectangles reflects the p-value of the respective enriched term among all enriched ones.
